# Complete Genome Sequence of *Streptomyces* sp. Strain SHP22-7, a New Species Isolated from Mangrove of Enggano Island, Indonesia

**DOI:** 10.1128/MRA.01317-18

**Published:** 2018-11-21

**Authors:** Ira Handayani, Shanti Ratnakomala, Puspita Lisdiyanti, Fahrurrozi, Wien Kusharyoto, Mohammad Alanjary, Regina Ort-Winklbauer, Andreas Kulik, Wolfgang Wohlleben, Yvonne Mast

**Affiliations:** a Department of Microbiology/Biotechnology, Interfaculty Institute of Microbiology and Infection Medicine, Faculty of Science, Eberhard Karls University of Tübingen, Tübingen, Germany; b Research Center for Biotechnology, Indonesian Institute of Sciences (LIPI), Cibinong, Indonesia; c Applied Natural Products Genome Mining, Interfaculty Institute of Microbiology and Infection Medicine, Faculty of Science, Eberhard Karls University of Tübingen, Tübingen, Germany; d German Center for Infection Research (DZIF), Partner Site Tübingen, Tübingen, Germany; Louisiana State University

## Abstract

*Streptomyces* sp. SHP22-7 is a novel strain isolated from a mangrove sample on Enggano Island, Indonesia. Here, we present the 7.9-Mbp genome sequence of SHP22-7, which will provide insight into its natural compound biosynthetic potential.

## Abstract (precis)

*Streptomyces* sp. SHP22-7 is a novel strain isolated from a mangrove sample on Enggano Island, Indonesia.

## ANNOUNCEMENT

Enggano Island is one of the outlying islands of Indonesia, located in the southwest of Bengkulu City with the coordinates 5°31′13″ latitude and 102°16′0″E longitude. It is a desert island 100 km from Sumatra Island and unique in terms of its high number of endemic species and richness in biodiversity (1, 2). An expedition to Enggano Island in 2015, coordinated by the Indonesian Institute of Sciences (LIPI), disclosed many new species of plants (3), animals (4), and microorganisms (5, 6). One of the microbial species that was isolated from a mangrove soil sample obtained from Enggano Island during that expedition is Streptomyces sp. SHP22-7, a potential antibiotic producer. As a general basis for further studies on the antibiotic production capacity of SHP22-7, we present here its complete genome sequence and bioinformatic analysis results.

For genome isolation, SHP22-7 was cultivated for 2 days in 50 ml of R5 medium (7) at 30°C. Genomic DNA was extracted and purified using the Genomic-tip 100/G kit from Qiagen (catalog number 10243). The genomic DNA isolation procedure was carried out following the standard protocol provided by the manufacturer. For proper cell lysis, achromopeptidase (5 mg/ml; Sigma) was added to the cells. For genome sequencing, a 10-kb paired-end library was constructed, and sequencing was performed with the Pacific Biosciences RS II platform. The genome was assembled using Hierarchical Genome Assembly (HGAP) V.3.0 (8). Altogether, 201,312 filtered reads (*N*_50_, 8,282 bp) were assembled to a nucleotide draft sequence of 7,899,734 bp with a 6-fold coverage. The total genome consists of 146 contigs with an average G+C content of 72.20%. Genome annotation was performed with the NCBI Prokaryotic Genome Annotation Pipeline (PGAP) (9). We observed 4,602 coding sequences (CDSs), 63 tRNAs, and 18 rRNA genes on the SHP22-7 genome. Using RaxML Web servers (10), we found that SHP22-7 is closely related to *Streptomyces* sp. CC71 (11). A MASH (12) analysis against all RefSeq (13) genomes yielded average nucleotide identity (ANI) estimates, which indicate that SHP22-7 is 95% similar to Streptomyces sp. CC71. This was confirmed with JSpeciesWS tree (14) with an ANI of 97.91%. The ANI higher than 95% indicates that SHP22-7 belongs to the same species as Streptomyces sp. CC71, a strain isolated from a sediment sample of the Cuatro Cienegas Basin, which is an oasis in the Chihuahuan desert in the north of Mexico (11).

The genome sequence of SHP22-7 was analyzed for secondary metabolite-specific biosynthesis gene clusters (BGCs) using AntiSMASH 4.0 (15), which predicted 25 BGCs. Three of the BGCs matched known clusters for albaflavenone (16), desferrioxamine B (17), and ectoine (18) with 100% similarity. Another three BGCs showed >80% similarity to clusters encoding amicetin (19), hopene (20), and candicidin (21) biosynthesis. The remaining clusters putatively encode 3 polyketides, 3 nonribosomal peptides, 2 nonribosomal peptide-polyketide hybrids, 2 terpenes, 2 bacteriocins, 2 lanthipeptides, 1 siderophore, 1 melanin, and 1 indole. Overall, the genome sequence of SHP22-7 provides useful information to further explore uncharacterized secondary metabolites from that strain.

### Data availability.

This whole-genome shotgun project has been deposited at DDBJ/ENA/GenBank under the accession number QXMM00000000. The version described in this paper is version QXMM01000000. Raw sequencing data are available under SRA accession number PRJNA489221. The genes used for phylogenomic analysis are available at https://figshare.com/articles/concatMLST_fasta/7228880. Alignments were done using a concatenated supermatrix of all genes accessible at https://figshare.com/articles/mlst_aligned_zip/7229099. For all software, default settings were used.
